# Role of chemotherapy prior to orchiectomy in metastatic testicular cancer—is testis really a sanctuary site?

**DOI:** 10.3332/ecancer.2014.407

**Published:** 2014-02-27

**Authors:** B Vinusha Reddy, A Sivakanth, G Naveen Babu, Krishnamurthy Swamyvelu, YG Basavana Goud, BA Madhusudhana, Vasu Reddy Challa

**Affiliations:** 1 Department of General Medicine, M.S Ramaiah Hospital, Bengaluru, Karnataka 560054, India; 2 Kurnool medical college, Kurnool, Andhra Pradesh 518002, India; 3 Vijayawada, Andhra Pradesh 520001, India; 4 Department of Surgical Oncology, Kidwai Memorial Institute of Oncology, Bengaluru, Karnataka 560029, India

**Keywords:** primary chemotherapy, delayed orchiectomy, advanced germ-cell tumours, germ-cell tumours

## Abstract

A germ-cell tumour (GCT) of the testis is a chemosensitive tumour with high cure rates even in advanced disease. Radical inguinal orchiectomy is the initial procedure used to diagnose it which helps to risk-stratify these patients. However, in patients with life-threatening metastases, primary chemotherapy was attempted in a few studies, followed by delayed orchiectomy. The aim of this review is to study the histopathological findings of delayed orchiectomy and the retroperitoneal lymph node dissection (RPLND) specimens, to assess difference and concordance in response rates in histological types of GCTs in pathological specimens. Overall, 352 patients received initial chemotherapy followed by orchiectomy, and 235 of them had undergone RPLND. Delayed orchiectomy specimens had viable tumour in 74 (21%) patients, scarring/necrosis in 171 patients (48.5%), and teratoma in 107 (30.3%) patients. RPLND specimens had residual disease in 36 (15.3%) patients, scarring/necrosis in 100 patients (42.5%), and teratoma in 99 patients (42.3%). Patients with seminoma who underwent delayed orchiectomy had complete disappearance of tumour in 81.3% of cases, and in non-seminomatous GCT, it was 43.4%. These results raise the question of the existence of a blood–testis barrier in patients with advanced GCT and argue against the testis as a sanctuary site.

## Introduction

Testicular cancer is a relatively rare malignancy with an incidence of 1–1.5% of male neoplasms in western countries and an incidence of 0.9% of male neoplasms in India [1, 2]. With the advances in imaging, surgical techniques, radiotherapy, and chemotherapy for testicular cancer, there is a steady increase in the survival rate, with a cure rate of 95% in patients with testicular cancer [3, 4]. Radical inguinal orchiectomy helps in diagnosis, is curative in early disease, and helps in risk-stratification of these patients. The management of testicular germ-cell tumours (GCTs) is determined by histological finding in the orchiectomy specimen and the extent of disease at the time of diagnosis. However, all advanced GCTs require chemotherapy regardless of the histological findings. With the availability of experienced pathologists and availability of serum markers, the diagnosis of the type of GCT can be made without orchiectomy. The other main reason for performing an initial orchiectomy is that the testis is considered to be a sanctuary site. This may be true in normal testis, but many studies have shown the complete disappearance of the tumour after chemotherapy, really calling into question the existence of the blood–testis barrier (BTB) in the presence of GCT [5–17].

Bleomycin/etoposide/cisplatin had become the standard of care in management of testicular tumours since their introduction in 1970s [18]. Delayed orchiectomy after primary chemotherapy had been attempted in patients with life-threatening metastases. The other situation where an upfront orchiectomy becomes difficult is in an advanced carcinoma testis in a patient with cryptorchid testis where the testis cannot be identified separately. In addition, individual studies had shown varied results with some correlating the histology of orchiectomy specimen with the retroperitoneal lymph node dissection (RPLND) and some studies with discordance.

Hence, in this review, we would like to assess the response rates at the primary and secondary sites in patients with advanced GCT after initial chemotherapy. We would also like to review the literature on the final histological findings in patients who underwent delayed orchiectomy and RPLND.

## Materials and methods

The following electronic databases were used for the literature search: Medline (1950–2012) and the Cochrane database. Then, a search was performed using the following search terms: primary chemotherapy for GCTs of testis, delayed orchiectomy, and advanced GCTs. After identifying relevant articles, the abstracts were studied and full articles were retrieved when the information in the study was eligible for review. In addition, cross references and related articles featured in PubMed were used to search for more articles.

## Study selection criteria

Only articles published in English were considered. Single-case reports and reports with less than five cases were excluded from the study. The following parameters were assessed: response to chemotherapy at primary and metastatic site, the final histopathology at primary and in resected specimens of metastatic site. An attempt was made to contact the authors if there were incomplete data for the study. In the absence of a response from the authors, the study was omitted from the review.

## Results

**Figure 1.**
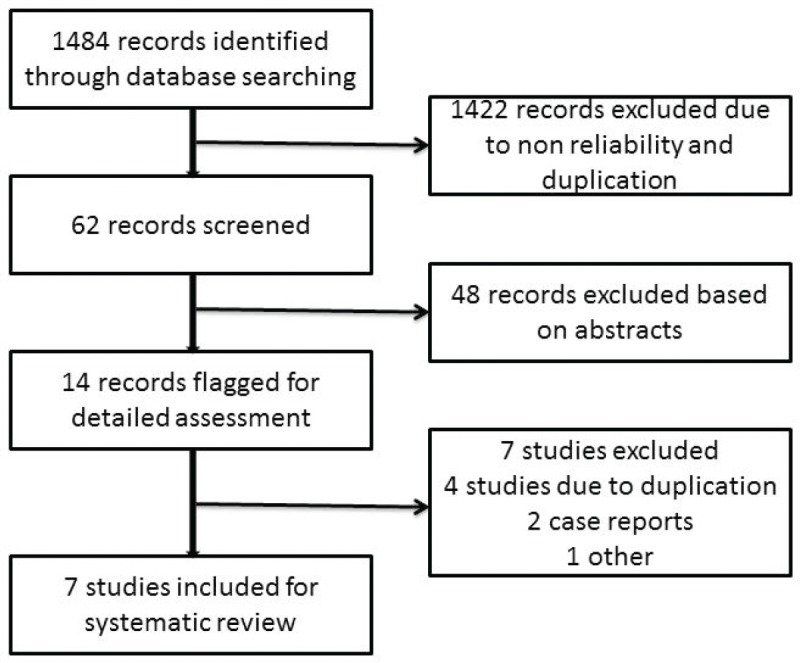
Flowchart showing the process of study selection for the systematic review.

After a literature search, 1484 records were obtained, of which 1422 records were excluded initially because of non-reliability and duplication (Figure 1). Of the available 62 records, 49 records were excluded based on the abstract. The full manuscripts of 13 articles were reviewed by the authors. Three of these were found to be continuations of previous studies [6, 7, 10], two were case reports [12, 15], and in one study, we could not get the complete details of the subjects in the study [14]. We reviewed seven eligible studies for the analysis as per the inclusion criteria. A total of 352 patients with advanced GCTs underwent primary chemotherapy followed by delayed orchiectomy. All of the studies used platin-based chemotherapy. The number of cycles given prior to orchiectomy varied in different studies. Histopathological examination of the delayed orchiectomy specimens revealed viable tumour in 21% of patients, scarring/necrosis in 48.5%, and teratoma in 30.3%. Among these 352 patients, 235 patients underwent RPLND (Table 1). We were not able to retrieve the data of the remaining 117 patients (even after an attempt to contact the authors) to find out the reason for not performing RPLND. Among 235 patients who underwent RPLND, 15.3% had residual disease, 42.5% had scarring/necrosis, and 42.2% had teratoma (Table 1).

On further subset analysis, in patients with seminomatous GCT alone, the response rate in the testis after orchiectomy showed necrosis or scar in 81.3% of patients (Table 2). In patients with non-seminomatous GCT after chemotherapy, necrosis or scar was present in 43.4% after delayed orchiectomy (Table 3). When histological correlation was studied for the primary and extragonadal site resected specimens in patients who underwent RPLND, the results varied from 48.1% to 84.6% (Table 4).

## Discussion

Various clinical observations had led to the concept of BTB. Even after completion of immune surveillance, sperms express new antigens, but they are recognised as self-antigens and no antibodies are produced against them. Hence, the testis is considered an immune-privileged site [15]. In addition, in childhood leukaemia, most relapses occur in the brain and testis, which further strengthens the idea of BTB [19–21]. Even in lymphomas, around 40–50% of relapses occur in the testis after chemotherapy [22]. Anatomically, the BTB is formed by the intersertoli junction, endothelial cells of blood vessels, and peritubular layer of myoid cells. There also exists a physiological barrier that is formed by the transport proteins, such as the P-glycoprotein and multidrug resistance protein-1, which efflux the drugs to interstitial space preventing the concentration of drugs in the cells [23, 24]. The mechanical BTB is maintained by the sertoli cells, which are under the influence of testosterone and follicle-stimulating hormone. A study in which methotrexate levels were compared with seminiferous fluid versus extracellular fluid (ECF) and vascular compartment found the difference in concentration levels of seminiferous fluid and ECF was 18–50, whereas it was 2–4 in comparison of ECF with vascular compartment [25].

The points against the concept of BTB are:, after the implementation of cisplatin-based chemotherapy for GCTs, there is a threefold decrease in the incidence of contralateral testicular tumours, supporting the hypothesis that the BTB may not be as significant as one would think in GCT [26, 27]. Hence, in early stage cancers, when the disease is confined to seminiferous tubules, the cancer cells may be protected by the BTB; but once the seminiferous tubules are damaged, they become devoid of BTB. In this review, 21% of patients have residual viable tumour after chemotherapy, 48.5% had scarring/necrosis, and 30.3% had teratoma. The largest study, by Leibovitch *et al* [11] from Indiana university, where 160 patients with non-seminomatous GCT (NSGCT) alone were evaluated, showed 43.7% with necrosis/scarring, teratoma in 31.2%, and residual viable tumour in 25% of cases. In other studies, where seminomas were included in the study, the risk of residual tumour in orchiectomy specimens varied from 0% to 66.7%. Ramani *et al* [16] found that all of the 13 patients with seminoma who underwent initial chemotherapy had scar tissue in the orchiectomy specimen.

Similarly, there are studies that compared the correlation of the histological response of the testis with the retroperitoneal lymph nodes. In our review, we had 42.5% of patients with scarring/necrosis, 15.3% of patients with residual tumour, and teratoma in 42.2% of patients. In a study by Simmonds *et al* [10], 11 of 13 patients had a histological correlation between orchidectomy and extragonadal metastases in resected specimens. However, in a study by Leibovitch *et al* [11], less than half the patients had histological correlation. Miller *et al* found a correlation in pathology in 61.9% of patients in testis and RPLN. The varied responses in various studies can be explained by the existence of BTB, tumour heterogeneity with variable responses to chemotherapy. Leibovitch *et al* evaluated salvage chemotherapy in 49 patients of 160 patients and found the response rates in the testis did not differ with primary or salvage chemotherapy, but there was higher rate of residual tumour in RPLN in patients who had received salvage chemotherapy. In addition, the patients who had complete pathological response had better survival when compared with patients with persistent GCT in RPLND specimens [11].

Oliver *et al* studied 78 patients who were given primary chemotherapy; 28 (36%) patients were considered for testis conservation (no orchiectomy), and 25 (32%) patients had no viable cancer on orchiectomy. Of the 28 patients considered for testis conservation, 26% relapsed between five and ten years and were later cured by orchiectomy. Testis conservation was most successful in stage I patients and in tumours less than 2 cm [14].

## Unanswered questions

Whether these patients can be considered for postchemotherapy surveillance or partial orchidectomy is still an area of investigation. A study by Kopecky *et al* [28], where postchemotherapy an ultrasound examination was performed, showed correlation of ultrasound with pathological examination. Whether this approach will be useful for patients with pure seminoma, where a teratomous component is very rare, is yet to be studied. Orchidectomy can be avoided if the size of hyperechoic lesion is less than 5 mm [16]. Another important area of investigation is whether PET-CT will be useful in seminoma patients with complete disappearance of tumour in testis, similar to the residual disease in retroperitoneum. Whether these responses will correlate with response in metastatic sites needs to be studied prospectively based on which orchiectomy specimen will help to decide on further management of residual mass in retroperitoneum. Finally, in patients who experience a disappearance of the tumour in the testis after chemotherapy, the feasibility of the testis-sparing approach or partial orchidectomy is yet to be studied in prospective trials.

## Conclusion

Although there are studies supporting the BTB, this review raises the clinical significance of this, with good responses occurring in the testis, similar to the RPLN sites. At the moment, orchiectomy is to be recommended in all cases, as residual disease was found in a certain per cent of cases in orchiectomy specimens on histopathological examination. We do not yet have enough evidence to draw a conclusion about testis preservation after primary chemotherapy in cases of metastatic testicular carcinoma. Further prospective studies are required to study the role of newer investigational modalities, the number of cycles of chemotherapy required prior to surgery, and a good protocol for follow-up of these patients.

## Figures and Tables

**Table 1.** Histopathological findings of delayed orchiectomy and RPLND specimens.

Author	Year	No. of patients	Type of GCT	Residual disease	Scarring/ necrosis	Teratoma	RPLND	Residual disease	Scarring/ necrosis	Teratoma
Calvo *et al* [5]	1983	5	5-NSGCT	3	2	0	2	1	1	0
Chong *et al* [8]	1986	16	11-NSGCT 5-Seminoma	4	9	3	1	1	0	0
Leibovitch *et al* [11]	1996	160	160-NSGCT	40	70	50	160	30	59	71
Ondrus *et al* [9]	2001	36	NA	3	18	15	24	1	13	10
Geldart *et al* [13]	2002	60	41-NSGCT/ Mixed, 18-Seminoma, 1-Unclassified	6	36	18	NA	NA	NA	NA
Ramani *et al* [16]	2008	33	20-NSGCT 13-Seminoma	6	21	6	6	0	2	4
Miller *et al* [17]	2012	42	35-NSGCT, 6-Seminoma, 1-Unknown	12	15	15	42	3	25	14
Total (%)		352		74 (21)	171 (48.5)	107 (30.3)	235	36 (15.3)	100 (42.5)	99 (42.2)

NSGCT: non-seminomatous germ-cell tumour.

RPLND: retroperitoneal lymph node dissection.

NA: not available.

**Table 2.** Published studies on primary chemotherapy in patients with seminomatous GCT.

Author (Year)	Number of patients with seminoma	Residual disease	Necrosis/scar
Chong *et al* (1986) [8]	5	1 (20%)	4 (80%)
Greist *et al* (1984) [7]^a^	1	NA	NA
Ng (1997) [12]	1^b^	0	1b
Geldart *et al* (2002) [13]	18	3 (16.7%)	15 (83.3%)
Ramani *et al* (2008) [16]	13	0	13 (100%)
Miller *et al* (2012) [17]^c^	6	4	2 (33.3%)
Total	43(44)^d^	8 (18.6%)	35 (81.3%)

a Details of this subgroup were not available.

b No orchidectomy was performed after complete response to chemotherapy detected on imaging.

c By personal contact.

d No details were available from one study by Griest whether there was residual disease or necrosis and hence analysis was done for 43 patients.

NA: Not available.

**Table 3.** Published studies on primary chemotherapy in patients with NSGCT.

Author (year)	Number of patients with non-seminoma	Residual disease	Teratoma	Necrosis/scar
Calvo *et al* (1983) [5]	5	3	0	2
Chong *et al* (1986) [8]	11	3	3	5
Leibovitch *et al* (1996) [11]	160	40	50	70
Geldart *et al* (2002) [13]	42	3	18	21
Ramani *et al* (2008) [16]	20	6	6	8
Miller *et al* (2012) [17]^a^	36	8	15	13
Total	274	63 (22.9%)	92 (33.5%)	119 (43.4%)

a By personal contact.

**Table 4.** Pathological correlation of orchiectomy specimen and extragonadal resected specimens in patients who underwent primary chemotherapy.

Author	Number of cases orchiectomy	Number of extragonadal masses resected	Histological correlation
Simmonds *et al* [10]	24	13^a^	11 out of 13 (84.6%) correlated (necrosis and/ or fibrosis in five, mature teratoma in four, and NSGCT in two)
Leibovitch *et al* [11]	160	160 (RPLND)	77 out of 160 (48.1%) correlated with orchiectomy
Ramani *et al* [16]	33	6 (RPLND)	5 out of 6 (83.3%) correlated with orchiectomy
Miller *et al* [17]	42	42 (RPLND)	26 out of 42 (61.9%) correlated with orchiectomy

a Out of 13, nine underwent RPLND, two underwent pulmonary metastatectomy, one underwent RPLND + pulmonary metastatectomy, and one underwent supraclavicular neckdissection.

RPLND: retroperitoneal lymph node dissection.

NSGCT: non-seminomatous germ-cell tumour.
